# *Tamarix articulata* extract offers protection against toxicity induced by beauty products in Hs27 human skin fibroblasts

**DOI:** 10.1371/journal.pone.0287071

**Published:** 2023-11-16

**Authors:** Abdullah M. Alnuqaydan, Faten M. Ali Zainy, Abdulmajeed G. Almutary, Najwane Said Sadier, Bilal Rah

**Affiliations:** 1 Department of Medical Biotechnology, College of Applied Medical Sciences, Qassim University, Buraydah, Saudi Arabia; 2 Chemistry Department, Faculty of Science, University of Jeddah, Jeddah, Saudi Arabia; 3 College of Health Sciences, Abu Dhabi University, Abu Dhabi, United Arab Emirates; 4 Iron Biology Research Group, Research Institute for Medical and Health Sciences, University of Sharjah, Sharjah, United Arab Emirates; King Abdulaziz City for Science and Technology (KACST), SAUDI ARABIA

## Abstract

The current study evaluates the cytotoxicity, mode of cell death and chemical analysis of selected beauty products and evaluation of the protective effect of *Tamarix articulata* (TA) extract against toxicity induced by beauty products in skin fibroblasts (Hs27). MTT and Crystal violet (CV) assays were used to determine the dose-dependent cytotoxic effects of beauty products against Hs27 fibroblasts. DNA fragmentation assay and annexin-V staining were conducted to determine the mode of cell killing induced by evaluated beauty products. Quantification of reactive oxygen species (ROS) and antioxidant enzyme levels were used to evaluate the oxidative stress. Chemical analysis and heavy metals were evaluated to determine beauty products. Pre-treatment with TA extract for different time points followed by time-dependent exposure with beauty products to assess the protective effect of TA extract in Hs27 cells was analyzed by MTT and CV assays. Owing to the presence of various harmful heavy metals such as arsenic (As), chromium (Cr), cadmium (Cd), nickel (Ni), and lead (Pb) in beauty products, our results revealed that all beauty products induce significant cytotoxicity over time (1, 4 h) in a dose-dependent (125, 250, 500 μg/mL) manner. DNA fragmentation assay, quantification of apoptosis by annexin-V staining, determination of ROS and antioxidant enzymes (CAT, GSH-Px and SOD) revealed that the induced cytotoxicity was caused by oxidative stress-mediated apoptosis. However, pre-incubation with a safe dose (50 μg/mL) of TA for different times (24, 48 h) followed by exposure to various doses (62.5, 125, 250, 500 μg/mL) of beauty products for different times (1, 4 h) revealed significant (*p≤0.05, **p≤0.01) protection against beauty product-mediated cytotoxicity. The effect was more pronounced for 1 h exposure to beauty products compared to 4 h. Our study demonstrates that the due to the presence of heavy metals in synthetic beauty products exhibit marked toxicity to skin fibroblasts due to oxidative stress-mediated apoptosis. However, the presence of abundant bioactive polyphenols with promising antiscavenging activity in TA extracts significantly nullifies cytotoxicity promoted by examined beauty products in skin fibroblasts (Hs27).

## Introduction

Skin is the largest, fast-growing organ and covers externally the whole human body to regulate body temperature, while fluid balance restricts the dangers posed by agents including chemicals, microbes, and protects against harmful sunlight-driven ultra-violet (UV) radiation. Besides protecting from various mechanical and harmful chemicals, skin provides thermoregulation, sensing stimulation, vitamin D synthesis as well as immune surveillance and provides personal identity thereby reflects social interference among individuals of a society [1]. Whereas skin is considered as one of the key personal identities, therefore, maintaining healthy skin to delay aging process of keratinocytes by cosmetic products opens the new door to explore anti-aging beauty or cosmetic products [1]. Composed of organic and inorganic ingredients, beauty products also have a low percentage of heavy metals as per international guidelines to act as preservatives, UV filters, antifungal and antibacterial agents [2]. While applied topically on the skin, the ingredients including heavy metals present in beauty products interacts with plasma proteins thereby circulated in the systemic circulation and finally reaches to liver where they undergo phase I and II biotransformation to their respective metabolites [2]. Hereafter, the metabolized products are either excreted through urine or accumulated within the organism [2]. However, consistent exposure and accumulation of harmful chemicals and radiation deteriorates not only skin morphology and physiology but also vital organs such as liver, kidneys which leads to progressive manifestations of aging [3]. Additionally, exposure to these products and radiation has serious implications such as lethal and prolonged absorption and life-threatening anaphylaxis (allergies) [4]. Apart from these outcomes, they could generate reactive oxygen species (ROS) in cutaneous cells and subsequently triggers the induction and activation of oxidative stress pathways [5]. This uncontrolled generation of ROS has been implicated in the pathogenesis of various skin diseases and often culminates in melanoma or skin cancer [6].

Beauty products comes in existence the date human civilization comes in the world [7]. Currently, synthetic cosmetic products and anti-aging products are the fastest-growing market around the world which claims that their active ingredients improve radiance, texture, skin tone and reduced wrinkles [8]. Recent reports claim that there is a 5% increase in beauty products annually [2], thus, suggest that anti-aging therapeutics is considered as forefront in cosmetic industry and dermatological research [9]. However, recent United States Food and Drug Administration (FDA) report suggests that more than 12,000 synthetic and related chemicals have been used in cosmetic products of which only less than 20% have proved to be non-toxic [10]. synthetic cosmetic products after absorption into the human body through various routes can have harmful effects such as disruption of endocrine and reproductive systems, functions as carcinogens and/or neurotoxicants. In effect, they pose a serious long-term threat to human beings [11].

Before the emergence of synthetic cosmetic products, natural ingredients derived from plant sources are used as beauty products in many societies. Consumers in the present scenario are more concerned about their health, thus demanding and advocating incorporation of bioactive natural constituents in cosmetic and beauty products for the betterment of their health [1]. Owing to the presence of abundant polyphenolic bioactive compounds that exhibit promising anti-scavenging activity with less toxicity and provides nutrients to skin cells [12], natural ingredients in plant-derived beauty products provide exogenous support of antioxidants and vitamins to skin cells. They neutralize ROS and other oxidative stress-related products to keep the skin cells healthy [13]. One such plant extract ingredients derived from the family *Tamaricaceae* is *Tamarix articulata* (TA). Phytochemical analysis revealed that the TA extract is rich in polyphenolic compounds which display promising pharmacological activities. TA is a halophytic plant abundantly found in the deserts of Saudi Arabia. Traditionally, TA has been used as a folk medicine by a tribal population–the Tafilalet in Morocco—against various ailments including skin diseases [14]. Owing to the presence of abundant polyphenolic compounds, TA extract showed promising antioxidant potential to scavenge ROS species generated during oxidative stress, as well as inhibits cell viability of various cancer cell models [14–17]. With these pharmacological activities in mind, the current study was designed to evaluate the protection offered by TA extract against the oxidative stress-mediated toxicity induced by beauty products in human skin fibroblasts. Our results suggest that the presence of abundant bioactive polyphenols with promising anti-scavenging activity of TA extracts significantly nullifies cytotoxicity promoted by beauty products in skin fibroblasts.

## Materials and methods

### Collection of plant material and preparation of extract

The plant material TA is scientifically well characterized belongs to clade: angiosperms, order: Caryophyllales, family: Tamaricaceae, genus: *Tamarix* and species: *Tamarix articulata* was collected in August 2019 from the deserts of Qassim province in the Kingdom of Saudi Arabia. A methanolic extract derived from the dry leaves was prepared as per the standard protocol published in our previous work [15]. After collection, the TA parts were first air-dried in the shade to remove the moisture completely. Using sharp blades, TA were chopped into small pieces, followed by grinding in a kitchen blender to produce a fine powder. After weighing 12 g of TA powder was added to 300 mL of 100% methanol and stirred constantly with magnetic bead for 5 days at room temperature. The mixture obtained was first filtered through cheesecloth to remove the bulk followed by filtration through a Whatman filter paper in an autoclaved glass beaker. The methanol (solvent) was completely evaporated from the plant extract mixture in a glass beaker by keeping the temperature of the hot plate at 45°C to avoid the degradation of heat labile compounds. After the complete evaporation of solvent, the fine powder of residue was left in the glass beaker was collected and stored at 4°C in stored vials for future experiments to evaluate the biological activities of the TA.

### Plant extract identification and characterization

TA plant parts (stem, leaves, and roots) were used in this study and were collected in December 2019 from the Qassim region of Saudi Arabia along with dried leaves found on the ground [15]. The identification and phytochemical analysis we recently published showed that major constituents of TA extract exhibits various pharmacological activities [14–17]. Further, phytochemical analysis suggests that TA extract is abundant in polyphenols (Table 1). The phytochemical analysis of the methanolic extract of TA by LC-MS analysis revealed that more than 200 compounds were identified (S1 Table in S1 File). The key phytochemicals identified from the methanolic extract of TA by LC-MS display anticancer activities against various cellular models and are summarized in (S1 Table and S1 Fig in S1 File).

**Table 1:** Major constituents of *Tamarix articulata* and their functions.

S. No.	Major Constituents	Functions	References
1.	Quinic acid	Anti-metabolic syndrome, radical scavenging, and cytotoxic activity	[18]
2.	Gallic Acid	Cardioprotective, antiangiogenic, anti-inflammatory, and anticancer activity, apoptosis-inducing potential	[19]
3.	Kaempferol	Hepatoprotective and anti-metabolic syndrome properties, anti-inflammatory, anticancer, and antiasthmatic activity	[20]
4.	Quercetin	Immuno-modulatory effect, anticancer, anti-inflammatory, and antiviral activity	[21]
5.	Tamarixetin	Anti-gelatinase and cardioprotective activity, arrests cell cycle and enhances bioavailability of other natural compounds	[22]
6.	Epicatechin gallate	Anti-metabolic syndrome (hypoglycaemic, antilipidemic, cardioprotective) activity, antioxidant potential	[23]
7.	Epiafzelechin	Anti-inflammatory, inhibits herpes virus, protects bones, and antiproliferative potential	[24]

### Liquid chromatography-mass spectrometry (LC–MS) metabolomic analysis and data processing

Liquid chromatography-mass spectrometry (LC–MS) was carried out as described in our recently published studies [15, 25, 26]. The LC–MS metabolomic analysis consisted of an AC-QUITY UPLC I-Class System (Waters Technologies, USA) coupled with a 6500 Qtrap (AB Sciex, Canada). A Zorbax XDB C18 column (2.1×150 mm. 3.5 μm) (Agilent, USA) was used for the chromatographic separation and kept at 40°C with a flow rate of 300 μL/min. The mobile phase consisted of A (0.1% formic acid in HPLC grade water) and B (0.1% formic acid in HPLC grade acetonitrile). The linear gradient elution was as follows: 2% B (from 0 to 2), 95% B (from 2 to 24), 95% B (held for 2 min), and then 4 min equilibration time. Electrospray ionization mass spectra (ESI-MS) were acquired in the positive (ES +), with an electrode voltage of 5500 V. The delustering potential was set at 90 V and the entrance potential was 10 V. Nitrogen was used as curtain gas (30 psi) and nebulizer gas on the MS. Spectra were collected with a mass range of 100–900 m/z. LC data files were converted to MZxml format using MS Convert (Pro-teoWizard 3.0.20270). MZ mine software (version 2.53) was used for the analysis of the data. After importing the data into the MZ mine, a minimum intensity cut-off of 1,000 was applied and the retention time was adjusted with a tolerance of 0.2 min. Adjusted peaks were then aligned into one mass list to facilitate identification and comparison. Compounds of interest were identified by using the KEGG Database in the finalized list based on m/z with a tolerance of 30 ppm.

### Beauty products sample collection

A total of six beauty products (lipstick products)—three beauty products from famous brands Huda Beauty, Revlon, and Maybelline (Saudi market–August-2019) and other three are unbranded—were collected from a local market. Beesline lipstick product served as the standard sample (made in Lebanon) which is tested and devoid of heavy metals and other deleterious chemicals. The ingredients of Beesline lipstick are carnauba wax, beeswax, wheat germ oil, carrot, sweet almond oil, honey, and vitamin E. The analyzed original brand lipstick samples were manufactured in Italy, USA, and Paris, while the unbranded lipstick products taken from the market have unknown manufacturer source. The prices of the original brand lipstick samples ranged from $28.00 to $11.37 USD per sample, while the unbranded samples ranged from $3.00 to $2.00 USD. All the samples were transferred to the laboratory for the analysis of their cytotoxicity and heavy metals.

All beauty product samples including standard sample (100 mg/mL) were dissolved in methanol and heated to 50 °C. The mixture is filtered in hot condition through 20 μm sterile cell culture filter membrane (Millipore) to obtain a clear sterile solution as filtrate. Each lipstick filtrate was diluted 100 times before the experiment in cell culture media to avoid any toxicity induced by methanol. The mixture was filtered by using sterile 20 μm filtered membrane to avoid any contamination to skin fibroblasts (Hs27 cells) used for experiments.

### Chemicals and reagents

The cell culture media Rosewell Park Memorial Institute (RPMI)-1640, antibiotics (penicillin-streptomycin), phosphate-buffered saline (PBS), and fetal bovine serum (FBS) were procured from Invitrogen. DNA fragmentation assay kit, annexin V FITC kit, ROS kit, GSH-Px, CAT, SOD and MTT were procured from Abcam. All other reagents and chemicals required for assays were purchased from Sigma-Aldrich.

### Cell culture

Human skin fibroblast cell line Hs27 cells were procured from ATCC. The cell line was maintained in culture media (Rosewell Park Memorial Institute (RPMI)-1640) supplemented with 10% FBS and 1% penicillin-streptomycin to avoid any bacterial contamination. The cells were grown and incubated in a 5% humidified CO_2_ incubator in sterile tissue culture T75 flasks. Hs27 cells were periodically checked for Mycoplasma contamination.

#### Cell treatments

Human skin fibroblasts Hs27 cells were cultured overnight to adhere to the surface of a flat bottom 96-well microplate at a density of 10^4^ cells per well and incubated in a 5% humidified CO_2_ incubator at 37°C. Next morning culture media was aspirated from each well of the microplate and replaced with fresh media containing varying doses (62.5, 125, 250, and 500 μg/mL) of beauty products, previously dissolved in cell culture media to decrease the concentration of methanol to avoid any toxicity effect induced by methanol for 1, and 4 h to determine cytotoxicity along with vehicle control to nullify any toxicity effect induced by methanol. However, for protection assay Hs27 cell after overnight incubation, cells adhered in wells of the 96-well microplate were preincubated with non-toxic doses (50 μg/mL) of TA extract for different times (24, 48 h). This was followed by exposure to varying doses (62.5, 125, 250, and 500 μg/mL) of beauty products for 1 h and 4 h to determine the level of protection against beauty product-induced cytotoxicity.

### Cell viability (MTT) assay

The cell viability of human skin fibroblasts (Hs27) was determined by MTT assay as per the standard protocol [27]. Briefly, 10^4^ Hs27 cells were seeded per well in the 96-microplate well which allowed the cells to adhere to the microplate’s bottom surface. The culture media in each well was aspirated and had fresh media added to it, containing varying doses of beauty products (62.5, 125, 250, and 500 μg/mL) and a non-toxic dose of TA extract (50 μg/mL) for different times (1, 4 h) and (24, 48 h), respectively. Following the completion of times, the wells containing cells incubated with 20 μl MTT dye (2.5 mg/mL) for 3–4 h at 37°C in 5% CO_2_ incubator. The formazan crystals formed during incubation were dissolved in DMSO (150 μl) by gentle vortex to ensure complete dissolution. The purple-colored solution formed in each well was measured at 570 nm by multiplate reader.

### Crystal violet (CV) staining for cell viability

Briefly, 10^4^ cells were seeded in each well of the 96-microplate well to determine cell viability utilizing CV assay. After overnight incubation to the cells and properly adhering to the bottom surface of the plate well, Hs27 skin fibroblasts were exposed to different concentrations of beauty (lipstick) products for varying times (1 and 4 h). The cells in each well were washed with PBS gently and then incubated with 50 μL of 0.5% CV solution in methanol for 10 min at room temperature. The wells of the 96-microplate well plate was washed with distilled water and then air-dried. After the plate wells were air-dried 50 μL distaining solution (33% acetic acid) was added. The blue color solution thus obtained was measured by multiplate reader at a wavelength of 570 nm.

### Sample preparation and analysis

The sample preparation for heavy metal analysis was done according to the standard method [28, 29] by using ICP-OES (Thermo-Scientific; ICAP 6000 Series). The standard operational conditions for the ICP-OES work were as follows: 1550 W-power; 15 L/min-plasma gas; 0.2 L/min-aux gas; 0.8 L/min-nebulizer; and 0.3 mL/min-sampling rate [30, 31]. To compare the quality control of original and fake brands HNO_3_ and HClO_4_ (65%–60%, Sigma-Aldrich) were adjusted so that they could digest the lipstick samples [7]. All the solutions were prepared in double-distilled water and required dilutions were created for analysis. Five heavy metals were investigated: As, Cd, Cr, Pb, and Ni. Every day, fresh calibration standards for each metal were prepared from the certified standard stock solution (High Purity Standards ICP-OES-68B Solution A, 100 mg/L in 4% HNO3) in the 0.5 to 10 ppm range.

### Apoptosis DNA ladder assay

The assay served to determine whether the cytotoxicity induced by beauty products was due to apoptosis or necrosis. Briefly, 0.5 ×10^6^ Hs27 cells were exposed to certain doses (250 and 500 μg/mL) of beauty products for 4 h and then harvested. DNA isolation of treated cells was done as per the manufacturer’s instructions stated in Apoptosis DNA Ladder Assay kit purchased from Abcam. After the isolation of genomic DNA from cells exposed to varying doses of beauty products, the samples from each treatment were resolved and analyzed by 2% agarose gel electrophoresis containing ethidium bromide (EtBr). After resolving was completed the agarose gel was analyzed by gel doc to generate an image.

### Detection of reactive oxygen species

Detection and quantification of ROS using DCFH-DA dye as described previously [32]. A stable non-fluorescent compound, DCFH-DA, is a permeable compound that, upon entering the cell, is further oxidized by ROS to form the fluorescent compound DCF, which is stable for a few hours. To determine and quantify ROS, 0.5 ×10^6^ cells were plated in each well of 6-well plates which contains sterile coverslips to allow cells grow on coverslips. Upon treatment with a beauty product along with positive control H_2_O_2_ and untreated control for 4 h, Hs27 cells were incubated with DCFH-DA (10 μM) at 37°C for 30 min in the dark. Next cells were washed in PBS and were first taken for absorbance at 485/535 nm and later accumulation of ROS in treated cells were viewed by fluorescence microscope (Leica DMI 3000B, Solms, Germany) for images.

### Apoptotic analysis

Detection and quantification of apoptotic cells were analyzed by using Annexin V and FITC apoptosis detection kit. As per the manufacturer’s instructions, briefly 0.5 ×10^6^ Hs27 cells were seeded in each well of a 6-well plate and allowed to adhere to the bottom surface of the well overnight at 37°C. The next morning, cells were exposed to beauty products and camptothecin (10 μM) as positive control alone, along with untreated control, for 4 h. Treated cells were harvested, washed with PBS, and subjected to Annexin V FITC/PI staining as per manufacturers protocol and were analyzed using BD Aria II flow cytometry.

### Determination of oxidative stress markers

For the measurement of oxidative stress markers, briefly 1.0 ×10^5^ Hs27 cells were seeded in each well of a 12-well plate and allowed to adhere to the bottom surface of the well overnight at 37°C, followed by treatment with beauty products for 4 h. After the treatment, Hs27 cells were harvested by cell scraper and washed with pre-cooled PBS followed by centrifugation and resuspension in distilled water. Resuspended cells were subjected to sonication for 15 seconds after every five second pause at 4°C followed by centrifugation at 14000 rpm for 10 min. The supernatant thus obtained was subjected to protein estimation by the Bradford method. After protein quantification from each beauty product treated cell sample aliquot were used to estimate glutathione peroxide (GSH-Px), superoxide dismutase (SOD), and catalase as manufactures protocol purchased from Abcam.

### Statistical analysis

All the experiments reported in the current study were done in triplicate. The results of the current study were calculated, processed by one-way ANOVA, and represents the mean of ± SEM. Statistical analysis was performed using GraphPad Prism software version 8, and a p-value of <0.01 was considered significant.

## Results

### Liquid chromatography-mass spectrometry (LC–MS) metabolomic analysis and data processing

Liquid chromatography-mass spectrometry (LC–MS) was carried out as described in our recently published studies [15, 25, 26]. The finding of phytochemical analysis of the methanolic extract of TA performed by LC-MS analysis revealed that more than 200 compounds were identified (S1 Table in S1 File). The key phytochemicals identified from the methanolic extract of TA by LC-MS display anticancer activities against various cellular models and are summarized in (S1 Table and S1 Fig in S1 File).

### Evaluation of cytotoxic effect and oxidative stress of beauty products on human skin fibroblasts (Hs27)

The cytotoxic effects of beauty products were determined by the MTT assay against the *in-vitro* skin cellular model (Hs27 cells). The MTT assay evaluated the cell viability against varying doses of beauty products. After seeding, Hs27 cells at a density of 10^4^ cells per well were put into 96-well microplates and allowed to adhere properly to the bottom surface overnight at 37°C in a 5% humidified CO2 incubator. Hs27 cells in the 96 wells were exposed to varying doses (62.5, 125, 250, and 500 μg/mL dissolved in culture media) of beauty products for a period of (1, 4 h) along with untreated control and a standard compound 16, which is known to exert an insignificant cytotoxic effect on human skin fibroblasts due to the absence of heavy metals and other toxic chemicals.

**Fig 1:**
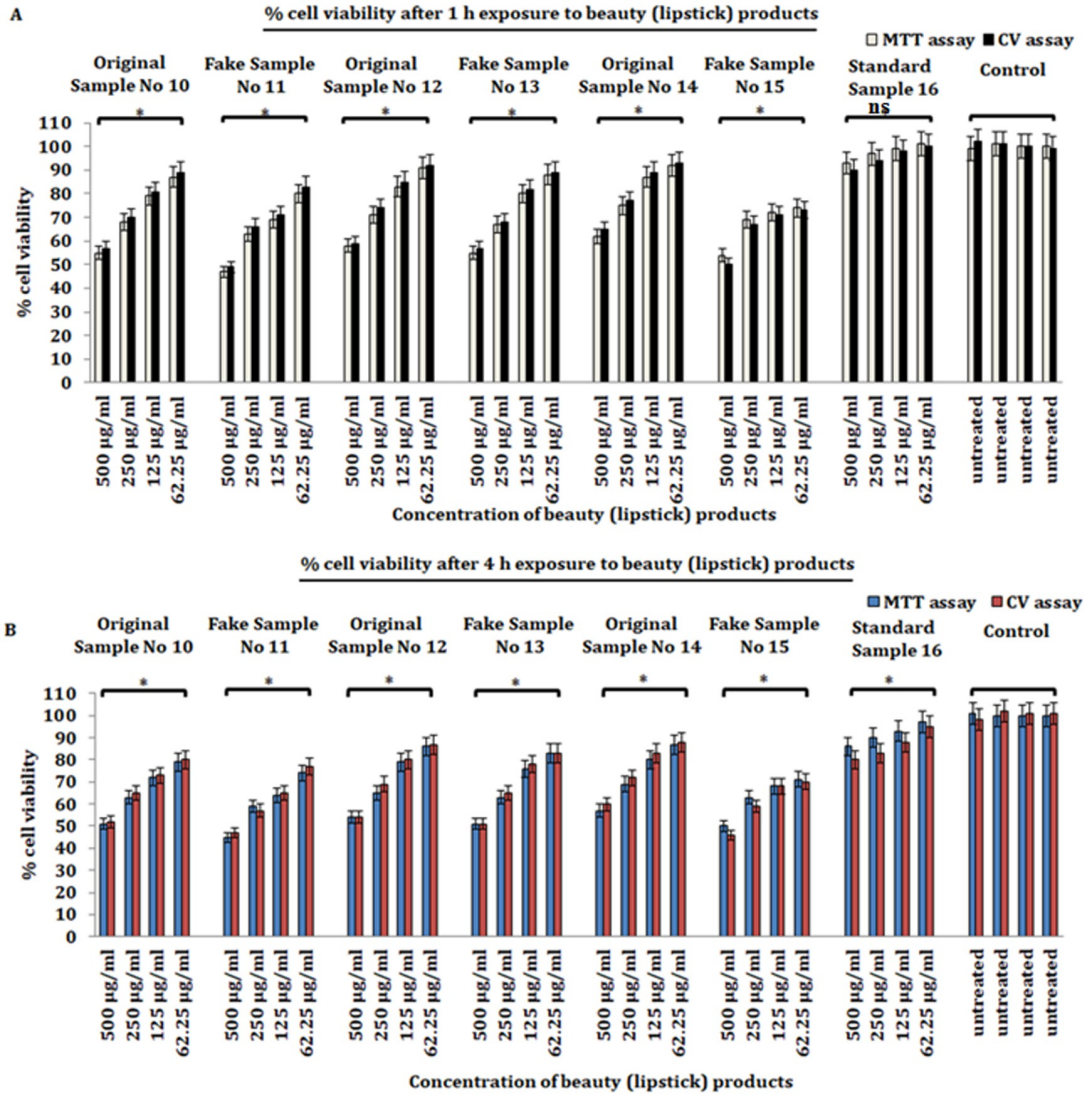
Evaluation of cell viability of Hs27 skin fibroblasts. **a**) Percent of cell viability of Hs27 skin fibroblasts exposed to varying concentrations (62.5, 125, 250, 500 μg/mL) of personal care products for 1 h. **b**) Percent cell viability of Hs27 skin fibroblasts exposed to varying concentrations (62.5, 125, 250, 500 μg/mL) of personal care products for 4 h. The data presented here is based on experiments done in triplicate and the mean value of ± SE. The p-value less or equal to 0.05 was statistically significant, *p ≤ 0.05.

Our MTT assay results demonstrate that significant cytotoxicity (*p* ≤ 0.05) was induced by beauty products at all doses and time points (1, 4 h) after the treatment when compared with untreated control and a standard compound 16. The latter revealed less cytotoxicity (Fig 1A and 1B). To verify the above MTT assay results, we undertook the crystal violet (CV) assay. Intriguingly, we observed a similar pattern with a significant percentage of cell cytotoxicity of Hs27 cells at all doses (62.5, 125, 250, and 500 μg/mL) of beauty products and time points (1, 4 h) as found earlier with the MTT assay. However, we did not observe any significant cytotoxicity effect of Hs27 cells when exposed to different doses of a standard compound at all time points when compared to untreated control (Fig 1A and 1B). Together, the cytotoxicity results suggest that: firstly, beauty products induce significant cytotoxicity to human skin fibroblasts Hs27; and secondly, might have induced and activated oxidative stress pathways to kill cells.

**Fig 2:**
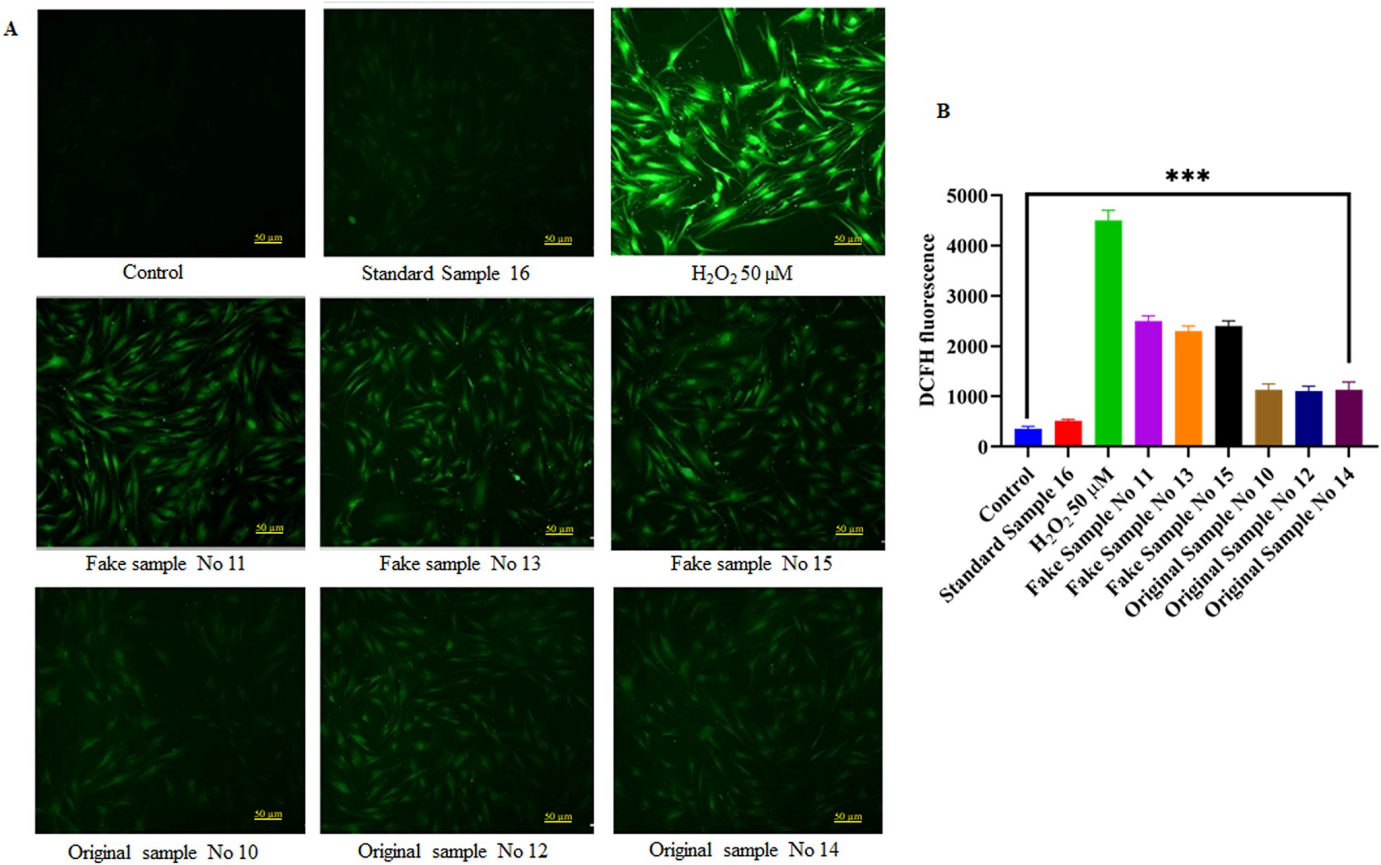
Beauty products treatment induces ROS-mediated oxidative stress in Hs27 cells. **a**) Representative micrographs of DCFHDA staining of Hs27 cells (20X) treated with beauty samples (Fake sample No. 11, 13, and 15 as well as original sample No. 10, 12, and 14), standard sample No 16 (Beesline lipstick product) along with positive control H_2_O_2_ 50 μM untreated control for 4 h. **b**) Bar diagram representing DCFHDA mean fluorescence intensity in beauty product treated Hs27 cells along with positive (H_2_O_2_ 50 μM) and untreated control cells. Scale bar is 50 μm. Graphed data is the mean ± SE of at least three independent experiments; ***p< 0.001.

**Fig 3:**
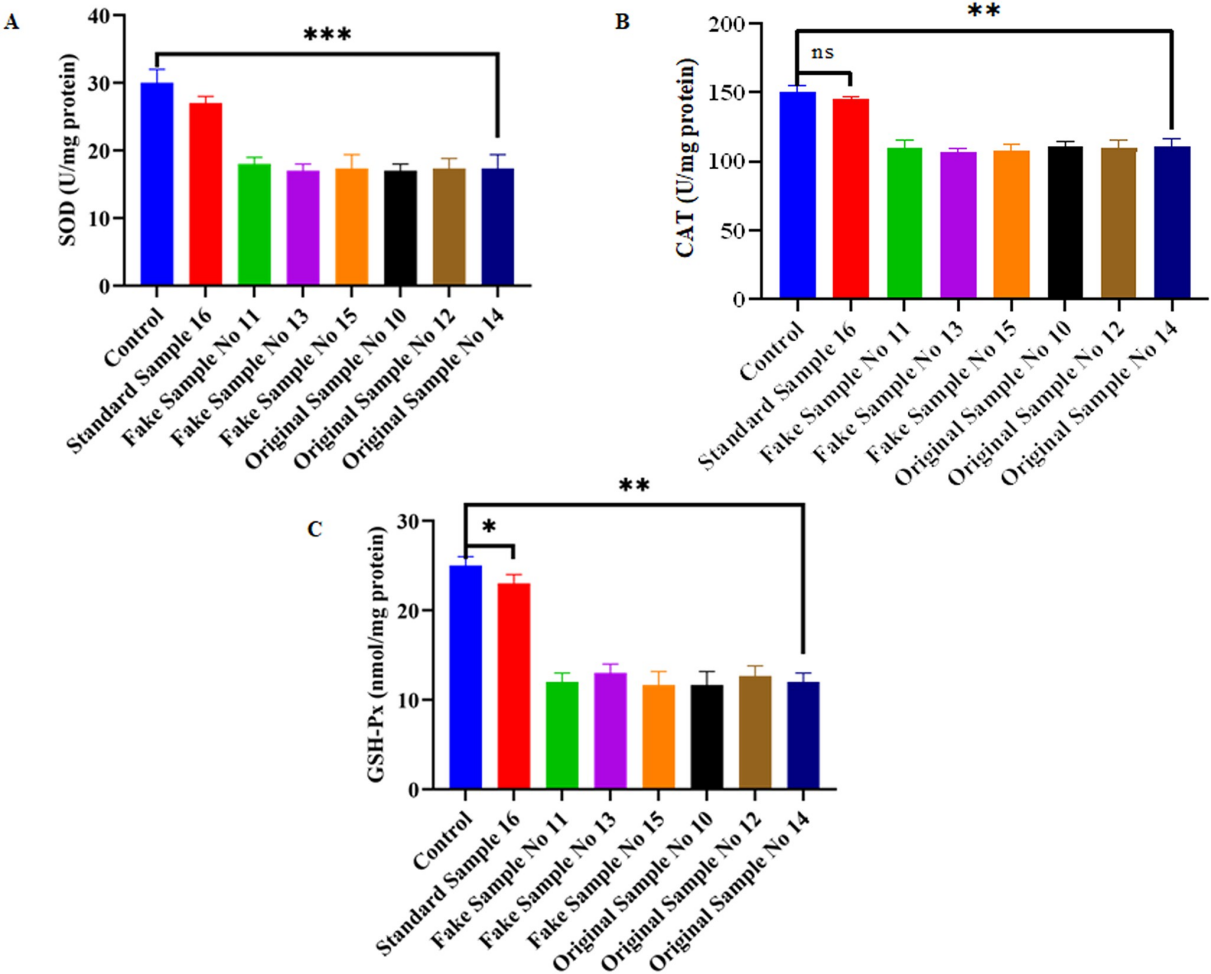
Effect of beauty products on oxidative stress markers (antioxidant enzymes) (SOD, CAT, GSH-Px) after treatment of Hs27 skin fibroblasts. **a)** Determination of superoxide dismutase (SOD) activity of Hs27 skin fibroblasts after the treatment with different personal care products for 4 h **b**) Determination of catalase (CAT) activity of Hs27 skin fibroblasts after the treatment with different personal care products for 4 h **c**) Determination of glutathione reductase (GSH-Px) activity of Hs27 skin fibroblasts after the treatment with different personal care products for 4 h. Graphed data is the mean ± SE of at least three independent experiments; *p< 0.05, **p< 0.01, ***p< 0.001.

We next sought to examine whether cell death observed in beauty product-treated Hs27 cells is linked to ROS-mediated oxidative stress. We evaluated the quantification of cellular ROS of Hs27 cells treated for 4 h with different beauty products along with H_2_O_2_ as a positive control and untreated control as a negative control. We observe significantly intense DCFHDA fluorescence of Hs27 cells exposed to beauty product samples compared to standard sample No 16 (Beesline lipstick product) and untreated cells. However, positive control H_2_O_2_-treated Hs27 cells were markely more intense DCFHDA staining when compared to control untreated Hs27 cells (Fig 2A and 2B). Further, we evalauted the oxidative stress markers such as SOD, GSH-Px and CAT in Hs27 cells treated with beauty products to authenticate above results. Our results demonstrated that a significant decrease of SOD, GSH-Px and CAT enzyme activity was observed when compared to standard beauty product (Beesline lipstick product) and untreated Hs27 cells (Fig 3A–3C). Collectively, these results suggest that beauty products augments ROS production and decreases the ezymatic activity of anti-oxidant markers which later drive oxidative stress-mediated cell death in Hs27-treated beauty product skin fibroblasts.

### Evaluation of cell death in Hs27 skin fibroblasts exposed to personal care products

**Fig 4:**
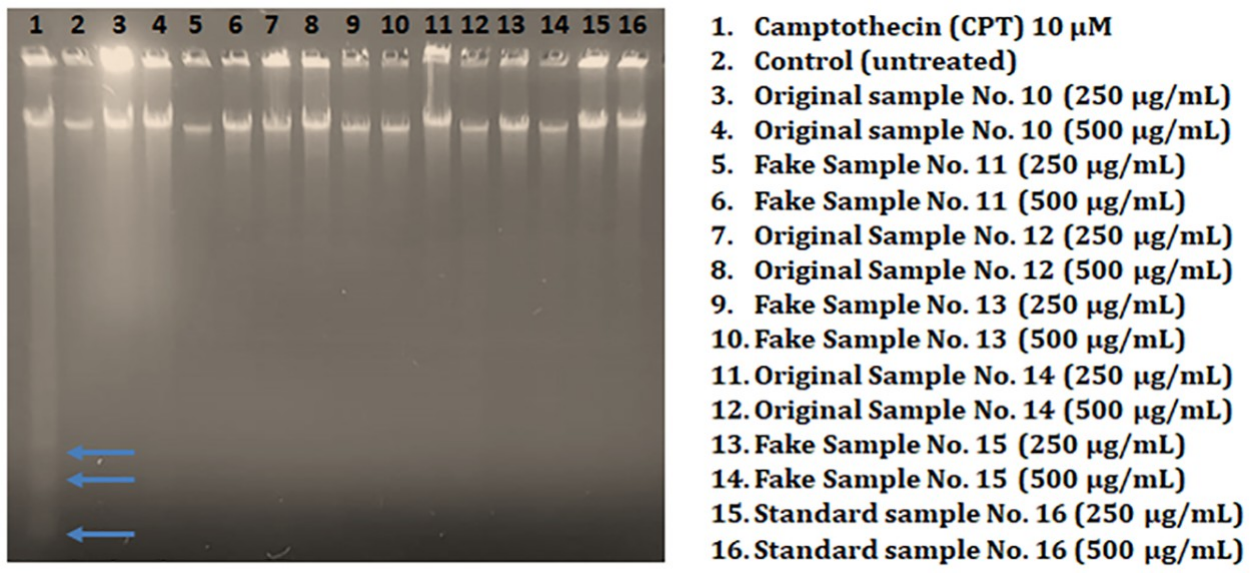
Determination of DNA fragmentation by agarose gel electrophoresis. Hs27 skin fibroblasts were exposed to varying doses of beauty (lipstick) products along with 10 μM camptothecin (positive control) to investigate the mode of cell death induced in Hs 27 cells. Arrows in the positive control lane showed DNA fragments indicate the apoptosis cell death, while the absence of DNA fragments in cells exposed to various doses of personal care products indicates that cell cytotoxicity induced by personal care products is due to necrosis.

To examine the mode of cell death induced by beauty products on skin fibroblasts Hs27. Following this, Hs27 cells were exposed to higher doses (250 and 500 μg/mL) of personal care products for 4 h along with untreated control and positive control camptothecin (10 μM) to analyze the mode of cell death in Hs27 cells. We executed DNA fragmentation assay by gel electrophoresis which is one of the most reliable methods for detecting apoptosis. Our results reveal that after resolving genomic DNA by electrophoresis in 1% agarose gel a smear pattern appeared, and no DNA ladder formation appeared in cell extract samples exposed to larger doses (250, 500 μg/mL) of beauty products. Nonetheless we observed a clear DNA ladder formation pattern in a lane of cell extract exposed to positive control camptothecin (10 μM) (Fig 4). These findings indicated that personal care product-induced cytotoxicity and could be due to oxidative stress-mediated apoptosis of skin fibroblasts.

**Fig 5:**
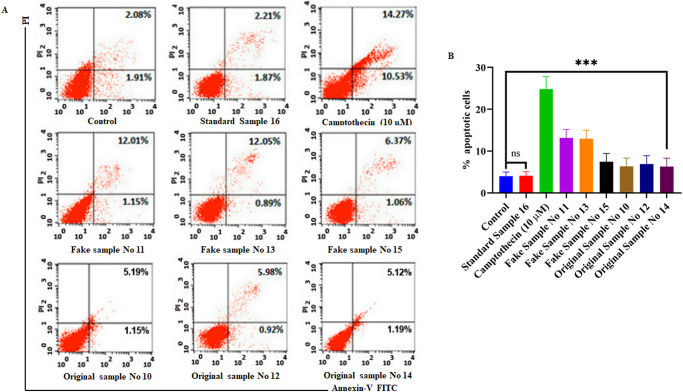
Beauty products treatment potentiates cell death via apoptosis in Hs27 cells. **a**) Annexin-V FITC and PI staining in Hs27 cells treated with personal care products for 4 h. **b**) Quantification of apoptotic cells from quadrants 2 and 3 by Annexin V FITC and PI staining. Graphed data is the mean value ± SE of at least three independent experiments; ***p< 0.001.

To further investigate the mechanistic evaluation of cell death in beauty product-treated Hs27 cells, we performed an annexin-V FITC assay for the quantification of apoptotic cells. A significant increase in apoptotic cell population was observed in Hs27 cells treated with beauty products (13.16%-fake sample 11, 12.94%-fake sample 13, 7.43%-fake sample 15, 6.34%-original sample 10, 6.90% original sample 12, 6.31%-fake sample 14) when compared with untreated control (3.99%) (Fig 5A and 5B). However, we did not find any significant population of necrotic cells. Thus these results suggests that Hs27 cell death induced by beauty products was due to apoptosis mechanism and this cell death could be due to the presence of heavy metals which drives ROS-mediated oxidative stress in Hs27 skin fibroblasts.

### Chemical and heavy metal analysis of the selected beauty products

Determining the various chemicals and heavy metals in selected beauty products collected from the Saudi market as discussed in the material methods section was conducted via the ICP-OES analysis system. Five heavy metals—arsenic (As), cadmium (Cd), chromium (Cr), lead (Pb), and nickel (Ni)—were performed using concentrated perchloric and nitric acid (1:4 ratio) to analyze these heavy metals. After analyzing the *beauty* products by the ICP-OES system, our results revealed that a small concentration of all five heavy metals was not only present in the fake beauty products (lipstick) but also in the branded ones (Table 2), yet the concentration was within the permissible limits as stipulated by the FDA. Interestingly, we did not find any heavy metal in the standard sample, meaning that the synthetic cosmetic products whether fake or branded (original) contain heavy metals that might induce collective toxicity to skin cells. This is well supported by significant cell killing of Hs27 skin fibroblasts by MTT assay as discussed in the previous results section.

**Table 2:** Concentrations (ppb) of heavy metals elements in beauty (lipstick) products.

Sample No.	Arsenic (As) mg/Kg	Cadmium (Cd) mg/Kg	Chromium (Cr) mg/Kg	Lead (Pb) mg/Kg	Nickel (Ni) mg/Kg
**Original 10**	ND*	2	15	22	27
**Fake 11**	ND	2	15	22	27
**Original 12**	ND	9	25	ND	26
**Fake 13**	ND	ND	ND	4	14
**Original 14**	ND	6	21	5	28
**Fake 15**	ND	1	19	5	23
**Standard 16**	ND	ND	ND	ND	ND

* ND: not detected

### TA extract protects against beauty products-mediated toxicity in skin fibroblasts

Owing to the presence of various polyphenols and flavonoid compounds that possess promising antioxidant properties, numerous plant products when combined with synthetic beauty products prevent the formation of ROS and other adverse effects on skin fibroblasts. We intended to determine whether TA extract could offer protection against beauty products-induced cytotoxicity to skin fibroblasts Hs27. To do this, we preincubated skin fibroblasts Hs27 with a safe dose (non-toxic) dose of TA extract (50 μg/mL) for different lengths of time (24, 48 h), followed by exposure to varying doses (62.5, 125, 250, and 500 μg/mL) of beauty products for different times (1, 4 h). This was done to evaluate how well TA protects against cytotoxicity induced by the examined products.

**Fig 6:**
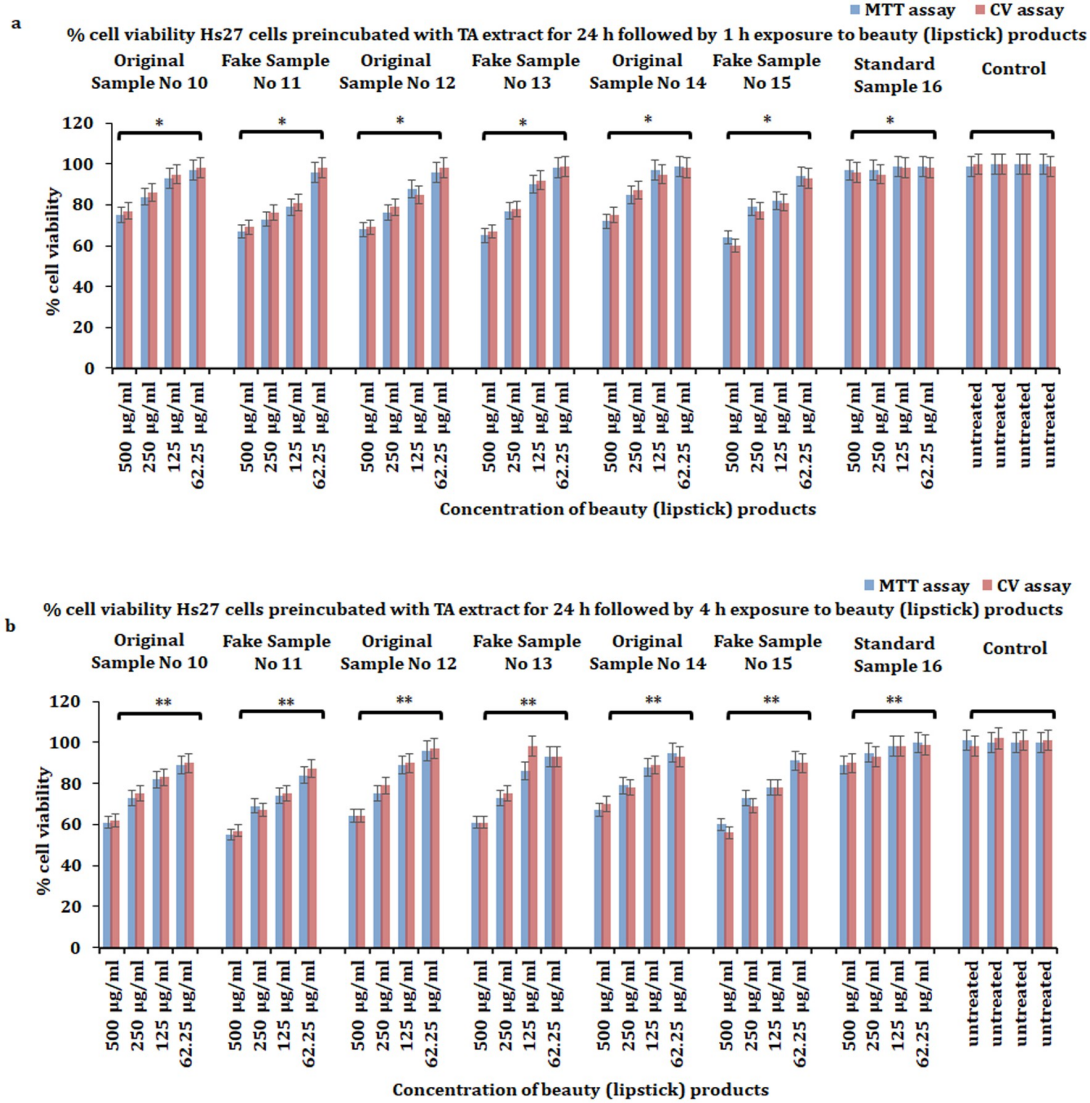
Protective effect of non-toxic dose (50 μg/mL) TA extract in Hs27 skin fibroblasts against the toxicity of beauty products. (**a**) Pre-treatment of Hs27 skin fibroblasts for 24 h with non-toxic dose (50 μg/mL) of TA extract, followed by treatment with varying doses (62.5, 125, 250, 500 μg/mL) of beauty (lipstick) products for 1 h to evaluate the percent cell viability. (**b**) Pre-treatment of Hs27 skin fibroblasts for 24 h with non-toxic dose (50 μg/mL) of TA extract, followed by treatment with varying doses (62.5, 125, 250, 500 μg/mL) of beauty (lipstick) products for 4 h to evaluate the percent cell viability. The data presented here is based on experiments done in triplicate and the mean value of ± SE. The p-value less or equal to 0.05 was considered to be statistically significant, *p ≤ 0.05, **p ≤ 0.01.

**Fig 7:**
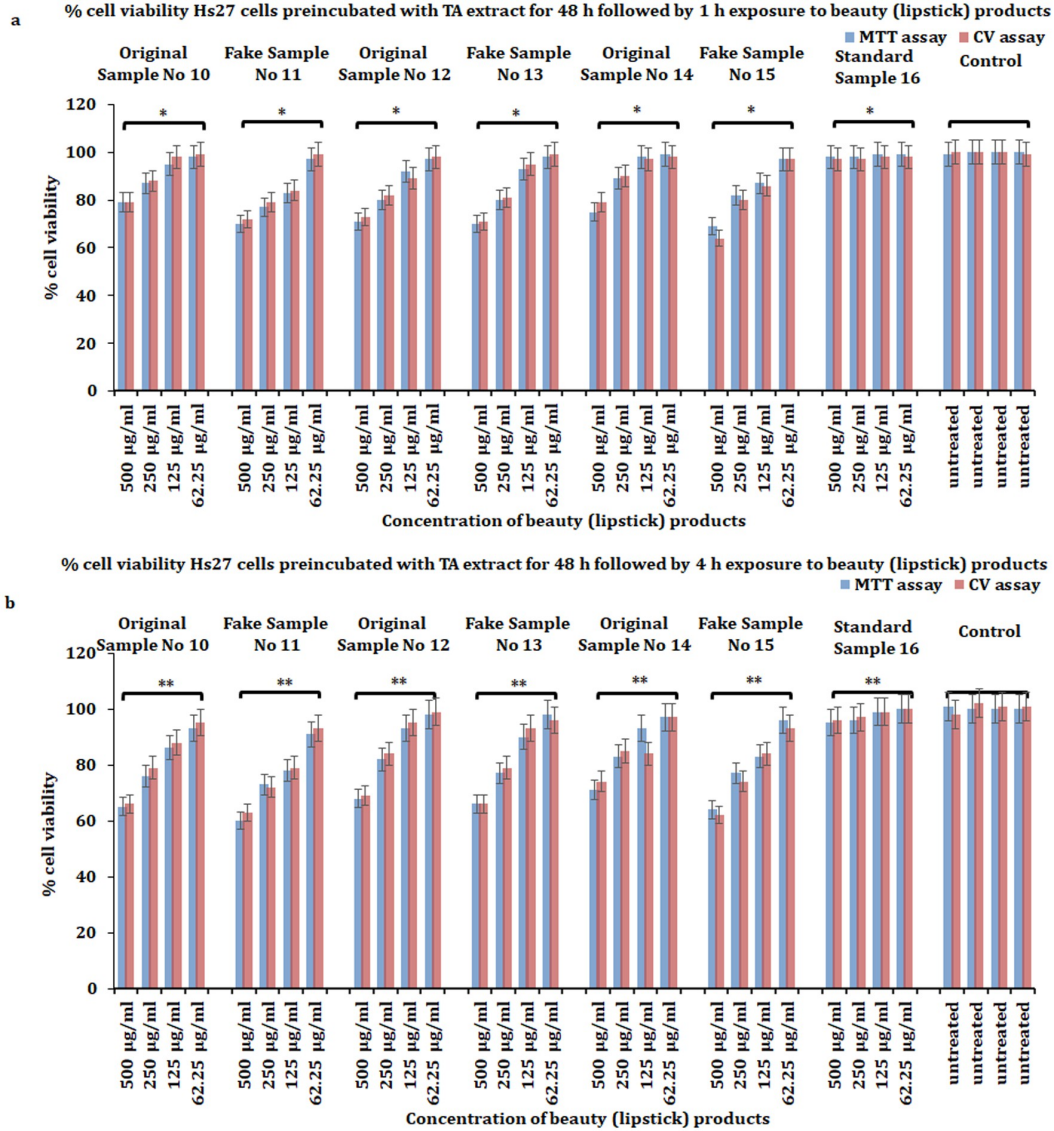
Protective effect of non-toxic dose (50 μg/mL) TA extract in Hs27 skin fibroblasts against the toxicity of personal care products. **a**) Pre-treatment of Hs27 skin fibroblasts for 48 h with non-toxic dose (50 μg/mL) of TA extract, followed by treatment with varying doses (62.5, 125, 250, 500 μg/mL) of beauty (lipstick) products for 1 h to evaluate the percent cell viability. **b**) Pre-treatment of Hs27 skin fibroblasts for 48 h with non-toxic dose (50 μg/mL) of TA extract, followed by treatment with varying doses (62.5, 125, 250, 500 μg/mL) of beauty (lipstick) products for 4 h to evaluate the percent cell viability. The data presented here is based on experiments done in triplicate and the mean value of ± SE. The p-value less or equal to 0.05 was considered to be statistically significant, *p ≤ 0.05, **p ≤ 0.01.

Our results revealed that TA extract significantly protects skin fibroblasts against the toxicity induced by all doses of beauty products at both times (1, 4 h) (Fig 6A and 6B). However, the protective effect against toxicity induced by examined products was more pronounced when cells were preincubated with a safe dose (50 μg/mL) of TA extract for 48 h (Fig 7A and 7B). Additionally, we extended the experiment by using preincubated skin fibroblasts Hs27 with a safe dose (50 μg/mL) of TA extract for 72 h, followed by exposure to beauty products for 1 and 4 h, to evaluate the protective effect against beauty product-mediated cytotoxicity. However, we did not find any increase in cell viability of skin fibroblasts after preincubation for 72 h. The reason might be the extortion of culture media for 72 h. Together, these results suggest that the TA extract can protect against the toxicity induced by beauty products, and it is most effective when skin fibroblasts are preincubated for 48 h with TA extract.

## Discussion

Exposure to numerous environmental pro-oxidants such as pollutants, radiation, chemicals, drugs, and cosmetic products causes oxidative stress in human beings, specifically at the cellular level in various organs including the skin and liver [33]. These pro-oxidant agents are harmful because they interact with bio-membrane proteins and lipids, which induces lipid peroxidation and interacts with DNA to induce genotoxicity in cells [25]. The cosmetic product market is one of the fastest-growing industries in the world and it is estimated that by 2022 it will earn more than $430 billion [26]. On average men and women use 6 and 12 cosmetic products, respectively, every day in the United States [34]. More than 12,000 industrial and synthetic chemicals have been reported as used in the synthesis of cosmetic products. However, less than 20% of these chemicals are reported to have a safe toxicity profile and not much attention has been paid to the usage of cosmetic products derived from these synthetic chemicals [10]. Synthetic personal care products induce irritation, allergic rashes, and toxicity to skin cells by generating ROS through oxidative stress mechanisms. Excessive production of ROS by personal care products in skin fibroblasts causes a variety of skin diseases including melanoma and associated cancers [35]. Tamarix articulata is abundant in polyphenolic compounds which have potential antioxidant activities. Traditionally, the leaves of this plant have been used as folk medicine by a tribal population-the Tafilalet in Morocco and Arab countries against skin diseases [14–17]. The aim of the current study is to evaluate the toxicity profile against Hs27 skin fibroblasts of beauty products and their chemical analysis. To date the TA extract is not used for cosmetic products. Therefore, the current study evaluated the bioactive properties of TA extract by testing on skin fibroblasts cellular models, and we hope it could be added to cosmetic products in future after having safe toxicity profile in animal models and clinical trials. Before the 1960s beauty products and personal care products had a good safety record except for a few make-up products which contained toxic chemicals and heavy metals such as cadmium, lead and mercury which were lethal to human beings [36]. Since the 1960s numerous scientific reports revealed that cosmetic products contain significant intoxicants in the form of chemicals and heavy metals which produce long-lasting photo-allergic and inflammatory skin reactions, subsequently posing a serious threat to consumers. For the past decade, the safety profile and chemical composition of synthetic beauty products have attracted great attention for evaluating the toxicity effects induced by these products against in-vitro skin fibroblast models and in vivo animal models.

Therefore, we set out to evaluate the chemical composition, cytotoxicity induced by beauty products against Hs27 skin fibroblasts as well as ROS-mediated oxidative stress and associated cell death. Our results revealed that a significant amount of cytotoxicity was induced by examined products when Hs27 skin fibroblasts were exposed to higher doses of the beauty products for 1 and 4 h. Under normal circumstances skin fibroblasts have a good enough antioxidant system to neutralize ROS species [37]. However, consistent exposure to pro-oxidants induces ROS species in cells and could promotes oxidative stress induced cell death in skin fibroblasts [37]. Consistent with previous findings our results revealed that beauty products induce significant ROS production in skin fibroblasts compared with untreated control cells which later induce oxidative stress-mediated cell death in cells. Plant extracts abundant in polyphenolic compounds have promising antioxidant properties to nullify any oxidative stress effect-mediated by ROS, thereby allowing recovery of skin fibroblasts from pro-oxidants. Another striking feature of oxidative stress affects the level of antioxidant enzymes such as CAT, GSH-Px, SOD etc which together act as oxidative stress markers to scavenge any pro-oxidant activity. These enzymes together constitute an antiscavenging system thereby catalyzing biochemical reactions to neutralize ROS species induced during oxidative stress. Recent evidence suggests that during oxidative stress the level of antiscavenging system enzymes is significantly reduced in oxidative stress cells. Our results depict that upon treatment of Hs27 skin fibroblasts with beauty products there is a sharp dip in the levels of CAT, GSH-Px and SOD enzymes. To explain the low levels of antioxidant enzyme levels of CAT, GSH-Px and SOD observed upon exposure to beauty products. The reason could be short exposure (1 and 4 h) of Hs27 skin fibroblasts to beauty products. Furthermore, consistent with previous results, upon pretreatment of skin fibroblasts with TA extract for 24 and 48 h followed by treatment with beauty products for 1 and 4 h, a significant recovery of cell viability of skin fibroblasts was observed indicating that antioxidants present in TA extract swiftly neutralizing ROS species induced by heavy metals present in beauty products thus helps recovery of skin fibroblasts. Together, these results suggest that polyphenols present in phytochemical extracts have ability to reduce the pro-oxidant species induced by heavy metals present in beauty products thereby helps the keratinocytes to recover from oxidative stress which acts as a major driver to kill pro-oxidant keratinocytes.

To analyze the chemical analysis of the selected products, our results revealed that the cell-killing induced was due to the presence of a collective concentration of heavy metals in personal care products. Previous reports suggest that the toxicological data of some chemical compounds such as benzalkonium chloride and diazolidinyl urea induces cytotoxicity and activates ROS-mediated mitochondrial-dependent apoptosis in cells [38, 39]. Avobenzone, commonly used in various personal care products promotes cell killing in human trophoblasts cells by calcium-mediated depolarization of the mitochondrial membrane potential to activate intrinsic apoptosis [40]. To evaluate whether the products in our study promote cytotoxicity by the activation of apoptosis, DNA fragmentation analysis demonstrated there was faint ladder pattern of DNA fragments when Hs24 cell lysate samples exposed to different doses of beauty products were resolved by agarose gel electrophoresis. For further investigation quantification of annexin-v FITC staining of beauty products treated Hs27 cells undergo apoptosis, however, we could not observe necrotic population in Q1 quadrant after treating Hs27 cells with beauty products. This strongly suggests that the cell-killing of Hs27 fibroblasts by beauty products was due to apoptosis induced by ROS-mediated oxidative stress.

Chemical and heavy metal analysis is currently an important parameter for establishing the safety of cosmetic products [7]. Although heavy metals are present in trace amounts in cosmetic products, long-term usage and exposure to beauty products that contain heavy metals even in traces can accumulate and pose a danger to vital organs such as the liver, kidneys, etc [41]. Some heavy metals such as Cd, Ni, and Cr can act as carcinogens to induce different types of malignancies and are categorized as group 1 human carcinogens by the International Agency for Research on Cancer (IARC) [42]. Apart from causing malignancies, these heavy metals pose a greater risk of neurological and cardiovascular disorders [7]. Accumulation of As after entering through various routes into the body causes various problems such as malignancies in the gastrointestinal system, lungs, and urinary tract [43].

Intriguingly, our results revealed that negligible As was detected in beauty products investigated in the current study. Cd is another heavy metal found in beautyproducts and long-term exposure to these products causes serious damage to kidneys. The concentration of Cd in the samples ranged from 1–9 ppb which is well below the desired concentration [44]. Cr and its salts provide brightness to cosmetic products. Cr is very essential for regulating insulin function and helps in glucose metabolism [10]. In the current study we observed maximum concentration of Cr in branded samples as compared to fake samples but within the permissible range. Pb exists in various cosmetic products and accumulates in the body either by inhalation or oral ingestion [45]. Accumulated Pb disturbs the central nervous system during fetus development in pregnancy and disturbs nerve transmission by interfering with calcium channels [45]. Pb detection concentration in the current study was also within the permissible range in all beauty products. Despite all the heavy metals in the current study found in permissible concentrations, due to the collective presence of all heavy metals in single beauty products and long-term exposure, they might pose a serious threat to human beings by causing various types of disorders. Owing to the presence of heavy metals, our cell viability assay suggests that these heavy metals collectively cause toxicity in Hs27 skin fibroblasts.

Products contain natural ingredients consisting of plant extracts, fruits, etc., that are rich in polyphenols and terpenes which act as antioxidants. The primary function of these compounds is to neutralize free radical species such as ROS, RNS, etc., and maintain the cell integrity free from any oxidative stress [46]. Mechanistically, these polyphenolic antioxidants possess phenolic groups that modulate protein phosphorylation of bio-membrane proteins to attenuate lipid peroxidation. They do this by acting as chain-breaking free radical scavengers thereby inhibiting oxidative reactions [47]. Plant extracts are in great demand in the cosmetics industry. The primary functions of plant products in the industry are to provide active ingredients and offer protection against other ingredients which can induce oxidative stress in skin fibroblasts [16, 48].

Owing to the presence of abundant polyphenols and flavonoid compounds in TA extract, we intended to evaluate the protective effect of TA extract in Hs27 skin fibroblasts against the toxicity induced by beauty products. Our results demonstrated that preincubation with a non-toxic dose of TA for different periods of time (24, 48 h) followed by exposure to various doses (62.5, 125, 250, 500 μg/mL) of beauty products for 1 h and 4 h, indicated significant protection against beauty product-mediated cytotoxicity. The effect was more pronounced with 1 h exposure to beauty products when compared to 4 h. Together, these results suggest that the presence of abundant bioactive polyphenols with promising anti-scavenging activity in TA extracts significantly nullifies cytotoxicity promoted by personal care products in skin fibroblasts.

## Conclusion

This study has demonstrated that synthetic beauty products (lipstick products) exhibit significant cytotoxicity against in-vitro cellular model Hs27 fibroblasts. The toxicity to the in-vitro skin fibroblast model by synthetic products was due to the collective effect of traces of heavy metals present in beauty products. It is confirmed here that the cytotoxicity induced by the examined products was due to oxdative stress-mediated apoptosis. Additionally, we observe TA extract, which is rich in antioxidant compounds, offers a protective effect and neutralizes any toxicity of skin fibroblasts induced by beauty products. Owing to the presence of toxic chemicals and heavy metals that trigger various adverse effects on the skin and other vital organs, there is an urgent need to assess the safety of beauty products. This can be done by incorporating natural plant extracts into the formulations of beauty products to neutralize any adverse effects.

## Supporting information

S1 File (DOCX)

S1 Raw image (PDF)
