# Supplementary material for: Tamarix articulata extract offers protection against toxicity induced by beauty products in Hs27 human skin fibroblasts
Source: PLoS One. 2023 Nov 16;18(11):e0287071. doi: 10.1371/journal.pone.0287071 (PMC10653522; doi:10.1371/journal.pone.0287071)

**Table 1.** The phytochemical analysis of the methanolic extract of TA by LC-MS analysis reveals the identification of more than 200 compounds.

| **S. No** | **M/Z** | **RT** | **Compound** |
| --- | --- | --- | --- |
| 1 | 103.08 | 6.447481 | 3-Methylbutanoic acid;2-Methylbutyrate |
| 2 | 104.04 | 0.781022 | Pyruvate oxime |
| 3 | 105.12 | 16.5714 | Choline |
| 4 | 107.04 | 22.75023 | Aromatic aldehyde; D-Glycerate |
| 5 | 108.96 | 24.27732 | Bromoethane |
| 6 | 119.04 | 11.48668 | Succinic acid |
| 7 | 126.120003 | 1.72972046 | γ-Coniceine |
| 8 | 128.04 | 24.26456 | 5-Amino-4-imidazole carboxylate;1-Methyl-4-nitroimidazole |
| 9 | 130.080002 | 0.79923889 | L-Pipecolate |
| 10 | 131.04 | 6.248767 | Itaconate;(E)-Glutaconate |
| 11 | 133.08 | 20.91498 | (R)-2-Hydroxyisocaproate;6-Hydroxyhexanoic acid |
| 12 | 134.04 | 6.839831 | L-Aspartate |
| 13 | 135.12 | 24.28927 | p-Cymene |
| 14 | 136.08 | 8.631082 | 2-Phenylacetamide |
| 15 | 137.04 | 15.86743 | Hypoxanthine; Threonate |
| 16 | 139.08 | 6.450736 | 4-Hydroxyphenylethanol; Styrene-cis-2,3-dihydrodiol |
| 17 | 140.039993 | 7.61881435 | 4-Nitrophenol;2-Nitrophenol;3-Nitrophenol;3-Hydroxypicolinic acid |
| 18 | 142.08 | 8.676849 | Hypoglycin; Arecaidine |
| 19 | 145.08 | 13.84979 | trans-4-Hydroxycyclohexanecarboxylate |
| 20 | 146.039993 | 0.78391111 | α-Ketoglutarate |
| 21 | 146.16 | 0.787356 | Spermidine |
| 22 | 147 | 5.374421 | Flupropanate |
| 23 | 147.12 | 13.21838 | L-Lysine |
| 24 | 149.04 | 22.59839 | D-Arabinono-1,4-lactone |
| 25 | 151.08 | 4.124735 | Tolylacetate |
| 26 | 160.080002 | 0.81778333 | Indole-3-acetaldehyde |
| 27 | 161.039993 | 17.2915444 | 2-Oxoadipate |
| 28 | 162.119995 | 6.89406976 | L-Carnitine |
| 29 | 163.08 | 24.28231 | Methyl cinnamate; Safrole |
| 30 | 165.12 | 24.24879 | Jasmone |
| 31 | 189.12 | 11.11041 | Glycyl-leucine |
| 32 | 167.16 | 5.766924 | Robinobiose |
| 33 | 175.08 | 22.49876 | N-Formimino-L-glutamate |
| 34 | 177 | 9.841988 | 3-Chloro-cis,cis-muconate |
| 35 | 178.080002 | 9.75604286 | 4-Hydroxy-4-methylglutamate |
| 36 | 177.12 | 10.12313 | L-Cladinose; Metaldehyde |
| 37 | 181.08 | 8.720061 | D-Glucose |
| 38 | 189.119995 | 11.1007144 | Glycyl-leucine |
| 39 | 193.080002 | 5.79493623 | Carpacin;Myristicin |
| 40 | 197.160004 | 8.60402733 | Linalyl acetate;alpha-Terpinyl acetate |
| 41 | 199.08 | 7.810947 | L-Mimosine |
| 42 | 205.199997 | 9.61738333 | β-Caryophyllene |
| 43 | 207 | 26.1537176 | Chloroneb |
| 44 | 207.12 | 26.1053 | 1,4-Dimethylphenanthrene |
| 45 | 209.039993 | 7.79105238 | Fraxetin |
| 46 | 209.16 | 12.44344 | Ammodendrine |
| 47 | 211.080002 | 10.4657552 | Sedoheptulose |
| 48 | 219.119995 | 16.7337904 | N-Acetylserotonin |
| 49 | 224.16 | 8.699876 | Tigloidine |
| 50 | 225.119995 | 13.7993487 | Aspidinol |
| 51 | 228.119995 | 20.8984778 | Ametryn |
| 52 | **229.080002** | **1.01080798** | **Resveratrol** |
| 53 | 232.080002 | 6.13259678 | N-Acetyl-L-2-amino-6-oxopimelate |
| 54 | 233.160004 | 10.3468355 | Alantolactone |
| 55 | 241.08 | 0.891821 | (1R,6R)-6-Hydroxy-2-succinylcyclohexa-2,4-diene-1-carboxylate |
| 56 | 245.039993 | 7.47793282 | L-Fuculose 1-phosphate |
| 57 | 245.16 | 22.48843 | Anagyrine |
| 58 | 248.039993 | 8.71224279 | Pyridoxal phosphate |
| 59 | 249.12 | 3.233528 | 6-Hydroxymelatonin |
| 60 | 254.16 | 21.66069 | Fenapanil |
| 61 | **254.279999** | **21.6546583** | **Solenopsin A** |
| 62 | 256.079987 | 23.6897444 | Apigeninidin |
| 63 | 257.160004 | 23.6928028 | Chanoclavine-I |
| 64 | **265.2** | **24.29171** | **Oxymatrine** |
| 65 | 266.16 | 24.28768 | Brevicolline |
| 66 | 270.12 | 24.60784 | Acetochlor |
| 67 | 276.119995 | 2.23423865 | (5-L-Glutamyl)-L-glutamine |
| 68 | 277.079987 | 0.93720015 | Biotin sulfone |
| 69 | 282.119995 | 24.2929528 | O6-Methyl-2'-deoxyguanosine |
| 70 | 299.160004 | 11.7539109 | Ostruthin |
| 71 | 300.119995 | 12.1321502 | 2,3,9,10-Tetrahydroxyberbine |
| 72 | 300.96 | 15.56441 | Tolclofos-methyl |
| 73 | 302.04 | 15.56511 | 2-(3,5-Dichlorophenylcarbamoyl)-1,2-dimethylcyclopropane-1-carboxylic acid |
| 74 | 307.079987 | 9.30033418 | Leucocyanidin;Gallocatechin |
| 75 | 307.08 | 9.293607 | Leucocyanidin; Gallocatechin; Epigallocatechin |
| 76 | 308.16 | 5.040135 | Tebuconazole |
| 77 | 311.04 | 9.821766 | Diflubenzuron; Edifenphos |
| 78 | 311.160004 | 9.16593779 | Nafenopin |
| 79 | 313.08 | 18.69813 | Inosine-5'-carboxylate |
| 80 | 313.2 | 18.79439 | 2,4,6-Triphenyl-1-hexene |
| 81 | 313.32 | 18.65702 | Icosanoic acid |
| 82 | 314.04 | 10.93399 | Isazofos |
| 83 | 315.12 | 18.68262 | Dihydropteroate; Pyraclonil |
| 84 | **315.24** | **18.68325** | **Dronabinol; Cannabidiol; Cannabichromene** |
| 85 | 316.08 | 18.69064 | Fenbendazole S-oxide |
| 86 | 316.2 | 18.68546 | Belladine |
| 87 | 318 | 14.04215 | Phosmet |
| 88 | 319.08 | 11.8284 | Lecanoric acid |
| 89 | 320.16 | 18.29014 | Metconazole |
| 90 | 321.12 | 0.803094 | Mugineic acid |
| 91 | 322.079987 | 2.18447318 | β-Citryl-L-glutamate |
| 92 | 323.04 | 10.14008 | Digallate |
| 93 | 324.119995 | 8.44852717 | Stylopine |
| 94 | 324.96 | 11.66847 | Trichloroethanol glucuronide |
| 95 | 325.2 | 18.62262 | Affinine |
| 96 | 325.08 | 13.86467 | Sterigmatocystin |
| 97 | 326.16 | 20.01459 | Monocrotaline |
| 98 | 327 | 27.18485 | Fenuron |
| 99 | 327.24 | 27.23535 | 12 α-Methylpregna-4,9(11)-diene-3,20-dione |
| 100 | 328.08 | 18.00045 | Fluazifop |
| 101 | 328.2 | 9.572027 | Sethoxydim |
| 102 | 328.32 | 15.33696 | N, N-Dimethylsphing-4-enine |
| 103 | 329.04 | 11.85282 | Fluorodifen |
| 104 | 329.16 | 12.03259 | 1,2-Dehydroreticuline |
| 105 | 330.12 | 12.42584 | Lithospermoside |
| 106 | 331.08 | 13.81768 | 3,7-Di-O-methylquercetin; Cirsiliol; Tricin |
| 107 | 331.2 | 13.81691 | Carnosol |
| 108 | 336.119995 | 17.2204175 | Trifluralin;Befuraline |
| 109 | **337.08** | **5.901588** | **Dicumarol** |
| 110 | 337.200012 | 5.67431458 | Catharanthine; Tabersonine |
| 111 | 337.320007 | 8.4919533 | (13Z,16Z)-Docosadienoic acid |
| 112 | 338.040009 | 2.47067165 | Azafenidin |
| 113 | 338.88 | 10.74711 | 1,2,3,7,8-Pentachlorodibenzofuran |
| 114 | 339.12 | 7.357424 | Glyceollin I; Glyceollin II |
| 115 | 341.04 | 24.88731 | Diclofop methyl |
| 116 | 342 | 24.85467 | Bifenox |
| 117 | 342.12 | 20.51962 | Protodeoxyviolaceinic acid |
| 118 | 346.079987 | 12.0925351 | Thiamine monophosphate |
| 119 | 349.200012 | 11.5365785 | Gibberellin A53 |
| 120 | 350.160004 | 2.21731399 | Riddelline |
| 121 | 353.04 | 11.55965 | Petunidin |
| 122 | 353.160004 | 12.090492 | Palmatine |
| 123 | 355.2 | 26.82419 | Vincamine; Yohimbine; Stemmadenine; |
| 124 | 355.32 | 27.48676 | 22-Oxodocosanoate |
| 125 | 356.16 | 27.42916 | S-Adenosylmethioninamine |
| 126 | 357.12 | 24.57235 | Gentiopicrin |
| 127 | 357.24 | 24.60749 | Arachidonyltrifluoromethane |
| 128 | 359.04 | 12.91192 | Triflumuron |
| 129 | 360.12 | 9.024405 | Isopenicillin N |
| 130 | 360.959991 | 2.27723168 | Chlorthiophos |
| 131 | 363.12 | 12.82633 | Chelirubine; Catalpol |
| 132 | 364.079987 | 10.812962 | Flufenacet |
| 133 | 365.04 | 0.799356 | Xanthosine 5'-phosphate |
| 134 | 365.160004 | 0.792625 | Gibberellin A8 |
| 135 | 367.08 | 5.559117 | Salicin 6-phosphate |
| 136 | 367.200012 | 5.68378438 | 16-Methoxytabersonine; Hirsuteine |
| 137 | 368.040009 | 9.69718675 | Anilofos |
| 137 | 370.08 | 14.05884 | S-(2-Chloroethyl) glutathione |
| 138 | 371.160004 | 6.76877846 | Bursehernin |
| 139 | 373.08 | 10.12554 | 2-O-Caffeoylglucarate |
| 140 | 373.200012 | 7.36471102 | Biocytin |
| 141 | 375.119995 | 23.1907687 | Secologanate |
| 142 | 375.12 | 13.1768 | Portulacaxanthin II;Swertiamarin;Geniposidic acid;Gardoside |
| 143 | **377.16** | **8.95533** | **Ailanthone** |
| 144 | 377.28 | 19.44733 | 9-cis-10'-Apo-β-carotenal |
| 145 | 379.079987 | 0.82208611 | 7-Methylguanosine 5'-phosphate |
| 146 | 383.16 | 3.540126 | 2-Methoxyestradiol-17β 3-sulfate |
| 147 | 385.32 | 22.32738 | (8Z,11Z,14Z,17Z,20Z,23Z)-Hexacosahexaenoic acid |
| 148 | 387 | 8.48086861 | Sulfentrazone |
| 149 | 387.12 | 10.09797 | 1-O-Sinapoyl-β-D-glucose |
| 150 | 389.160004 | 10.6243039 | Visnadin |
| 151 | 390.959991 | 7.31033764 | 5-Phospho-α-D-ribose 1-diphosphate |
| 152 | 393.119995 | 6.13023356 | Macarpine |
| 153 | 395.16 | 15.13311 | Rotenone; Deguelin |
| 154 | 395.399994 | 17.3745676 | Ximenic acid |
| 155 | 397.32 | 12.56728 | δ-Tocotrienol |
| 156 | 398.160004 | 12.6067147 | Aureothin |
| 157 | 399.12 | 7.147485 | S-Adenosyl-4-methylthio-2-oxobutanoate |
| 158 | 401.28 | 7.685251 | Ophiobolin A |
| 159 | 403.2 | 9.7105 | Prednisolone acetate; Citreoviridin |
| 160 | 407.16 | 17.18432 | Asperlicin C |
| 161 | 409.2 | 9.246711 | Abyssinone V |
| 162 | 410.160004 | 3.91140291 | Linustatin |
| 163 | 411 | 11.26515 | Imibenconazole |
| 164 | 411.12 | 14.16161 | Nicosulfuron; Spirodiclofen |
| 165 | 411.24 | 17.67294 | 1-Palmitoylglycerol 3-phosphate |
| 166 | 411.36 | 18.69642 | 5-Dehydroavenasterol; Avenastenone |
| 167 | 412.320007 | 23.3697828 | Cyclopamine |
| 168 | 413.04 | 22.43382 | Imazosulfuron; Pyraflufen-ethyl |
| 169 | 413.040009 | 22.4248369 | Pyraflufen-ethyl |
| 170 | 414.96 | 17.5999 | Flusulfamide |
| 171 | 415.08 | 19.1103 | (7R)-7-(5-Carboxy-5-oxopentanoyl) aminocephalosporinate |
| 172 | 415.320007 | 25.4925863 | Yamogenin |
| 173 | 417.12 | 11.14687 | Daidzin; Frangulin A; Puerarin |
| 174 | 418.200012 | 13.5014327 | Casimiroedine |
| 175 | 423 | 14.1758789 | 5-Fluorouridine diphosphate |
| 176 | 423.119995 | 22.6974831 | Plicatic acid |
| 177 | 425.279999 | 17.1062564 | α-Phocaecholic acid |
| 178 | 427.2 | 12.38413 | Abscisic acid glucose ester |
| 179 | 428.040009 | 12.3795444 | Adenosine 3',5'-bisphosphate |
| 180 | 428.16 | 12.38573 | Vicianin |
| 181 | 429.12 | 23.93726 | ε-Rhodomycinone; Quizalofop-P-tefuryl |
| 182 | 429.24 | 23.93274 | Nummularine F |
| 183 | 430.320007 | 21.7180979 | Imperialine |
| 184 | 437.519989 | 17.0830779 | Hentriacontane |
| 185 | 438.23999 | 9.0921256 | Lunarine |
| 186 | 441.12 | 10.95042 | Hallactone B |
| 187 | 441.36 | 16.10699 | Soyasapogenol C; Oleanolic aldehyde |
| 189 | 443.160004 | 0.95589167 | Mallotochromene |
| 190 | 447.12 | 4.350199 | Cephamycin C; Glycitin |
| 191 | 453.12 | 26.06561 | Cinchonain 1a |
| 192 | 453.36 | 25.52124 | Phylloquinol |
| 193 | 455.160004 | 14.5180328 | ε-Viniferin |
| 194 | 458.16 | 20.93682 | Amygdalin |
| 195 | 460.200012 | 8.64017559 | 5-Methyltetrahydrofolate |
| 196 | 461.160004 | 9.34915583 | Paeonolide |
| 197 | 461.279999 | 21.0038789 | Sophoranone |
| 198 | 461.399994 | 9.51068431 | Protopanaxadiol |
| 199 | **462.959991** | **13.8378296** | **Quercetin 3,3'-bissulfate;Quercetin 3,4'-bissulfate** |
| 200 | 463.079987 | 14.0931398 | Luteolin 7-O-glucuronide |
| 201 | 463.200012 | 16.7546761 | Plicamine |
| 202 | 467.160004 | 22.6358392 | Agnuside |
| 203 | 467.279999 | 20.3436707 | Cephaeline |
| 204 | 471.36 | 21.25501 | Gypsogenin |
| 205 | 473.399994 | 22.5188859 | α-Tocopherol acetate |
| 206 | 474.12 | 16.33285 | CMP-N-trimethyl-2-aminoethylphosphonate |
| 207 | 485.28 | 25.71086 | Stigmatellin |
| 208 | 486.96 | 5.876778 | 5-Fluorodeoxyuridine triphosphate |
| 209 | 487.200012 | 23.1899191 | Rutaevin; Haplodimerine; Nafenopin glucuronide |
| 210 | 489.36 | 24.9554 | Asiatic acid |
| 211 | 489.12 | 9.603542 | CDP-choline |
| 212 | 492 | 10.758352 | dATP |
| 213 | 495.119995 | 23.7432148 | 5'-Methoxyhydnocarpin-D |
| 214 | 497.279999 | 23.6242253 | Absinthin |
| 215 | **517.2** | **22.54293** | **Rottlerin** |
| 216 | 517.320007 | 23.5029426 | Cucurbitacin D |
| 217 | 525.47998 | 22.7749935 | Retinyl palmitate |
| 218 | 541.200012 | 21.6297253 | Oleuropein |
| 219 | 553.44 | 24.15608 | Zeinoxanthin; β-Cryptoxanthin |
| 220 | 593.400024 | 24.1761293 | Santiaguine |
| 221 | 625.200012 | 13.1988915 | Verbascoside |
| 222 | 629.280029 | 23.81974 | Resiniferatoxin |

**Table 2.** The major compounds are encapsulated in Table 2 and their respective chromatograms were presented in Figure 1.

| **S. No** | **Major Compounds** | **Functions** | **References** |
| --- | --- | --- | --- |
| 1. | Solenopsin | Exhibits antiproliferative activity and inhibits PI3K/Akt driven angiogenesis | [30] |
| 2. | Ailanthone | Promotes apoptosis and autophagy by increasing the expression of miR-195 in leukemia cells | [31] |
| 3. | Dicumarol | Exhibits promising antiproliferative, and induces ROS-mediated mitochondria-dependent apoptosis in breast cancer cells | [32] |
| 4. | Dronabinol | Promotes antiproliferative activity and induces apoptosis by cannabinoid receptor (CB) ½ in leukemia cells | [33] |
| 5. | Oxymatrine | Induces apoptotic cell death and arrests G0/G1 cell cycle phase by blocking EGFR/PI3K/Akt/mTOR signaling in glioma cells | [34] |
| 6. | Quercetin | Immuno-modulatory effect, anticancer, anti-inflammatory, and antiviral activity | [35] |
| 7. | Resveratrol | Exhibits antioxidant, anti-inflammatory and antiproliferative activity, interferes in many signaling pathways and activates apoptosis in cancer cell models | [36] |
| 8. | Rottlerin | Antihypertensive, antiallergic and antifertility activities. Promotes antiproliferative activity by downregulating NF-kB and cyclin D1 expression. | [37] |

**Figure 1.** Chromatogram of key phytochemicals present in TA extract ( This figure showed data from our recently published work [18,20,21]).

1.Solenopsin


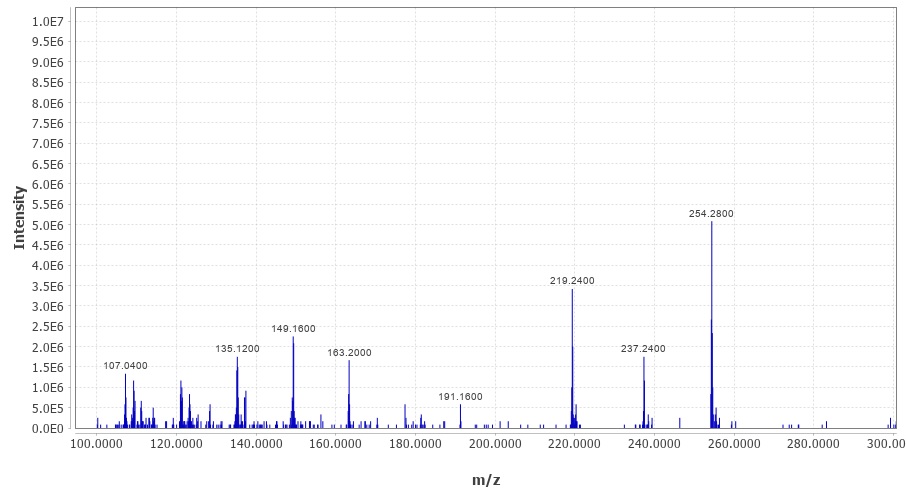


2. Ailanthone


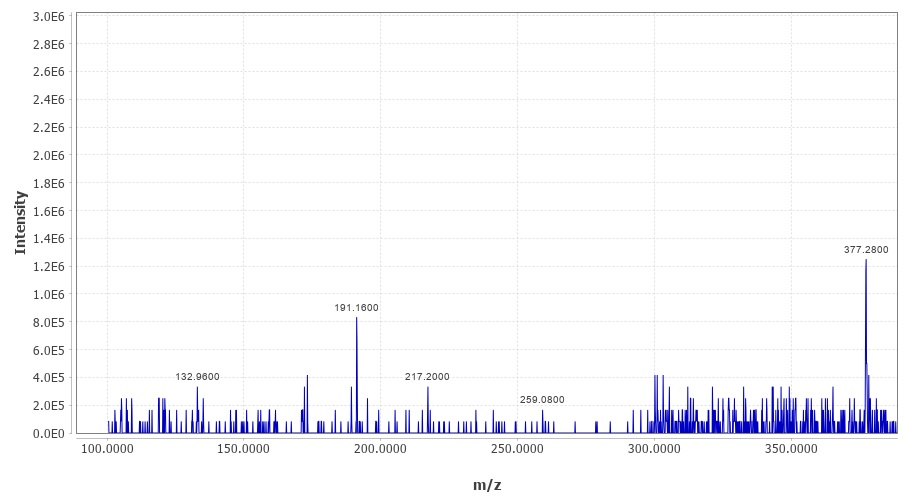


3. Dicumarol


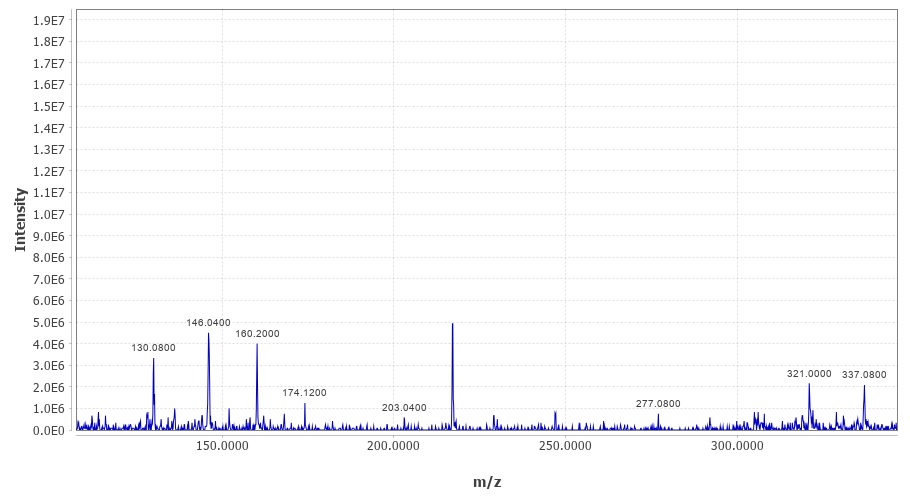


4. Dronabinol


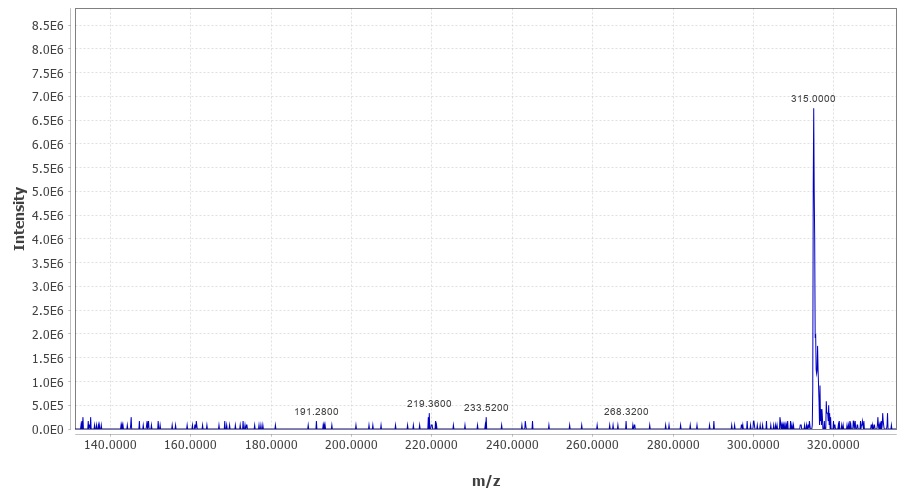


5. Oxymatrine


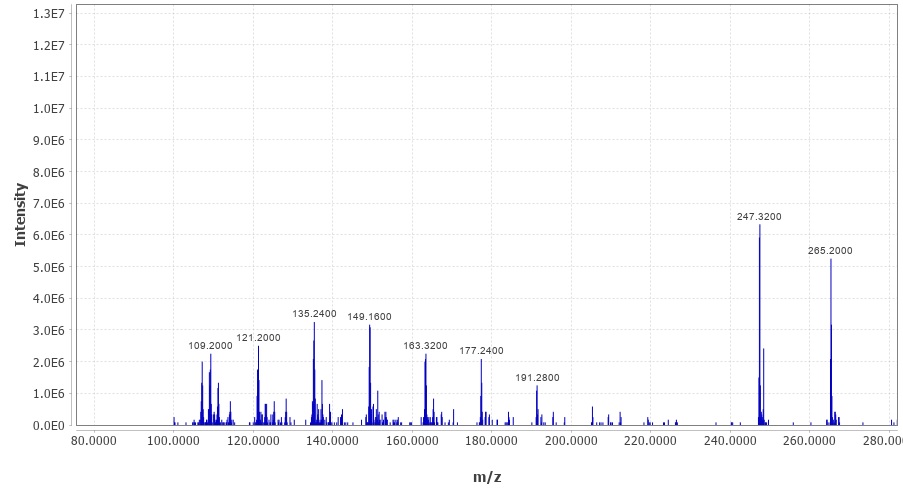


6. Quercetin


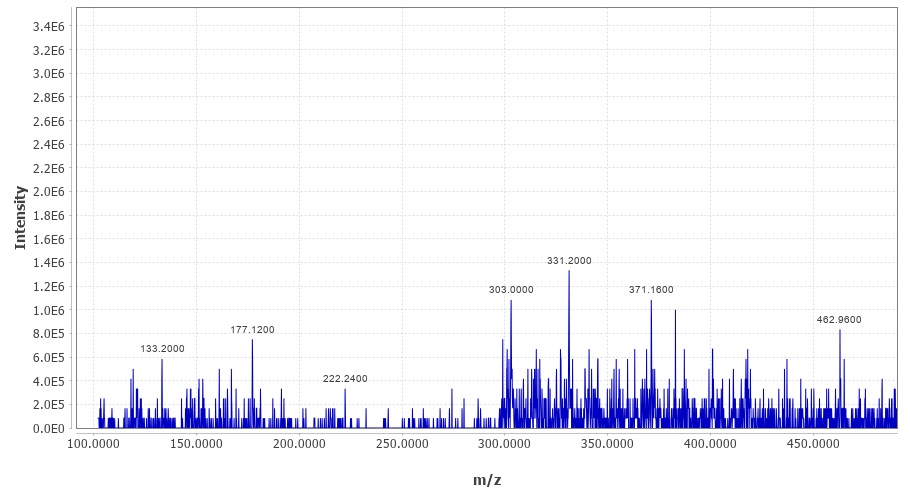


7. Resveratrol


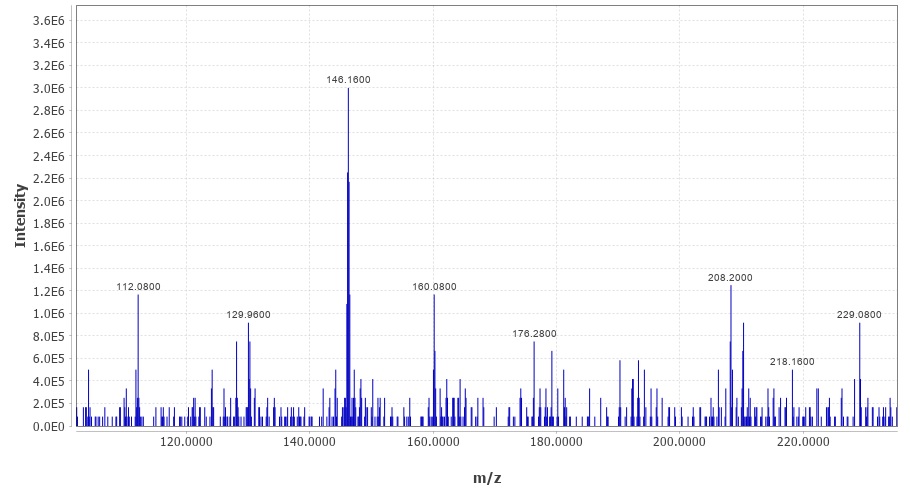


8. Rottlerin


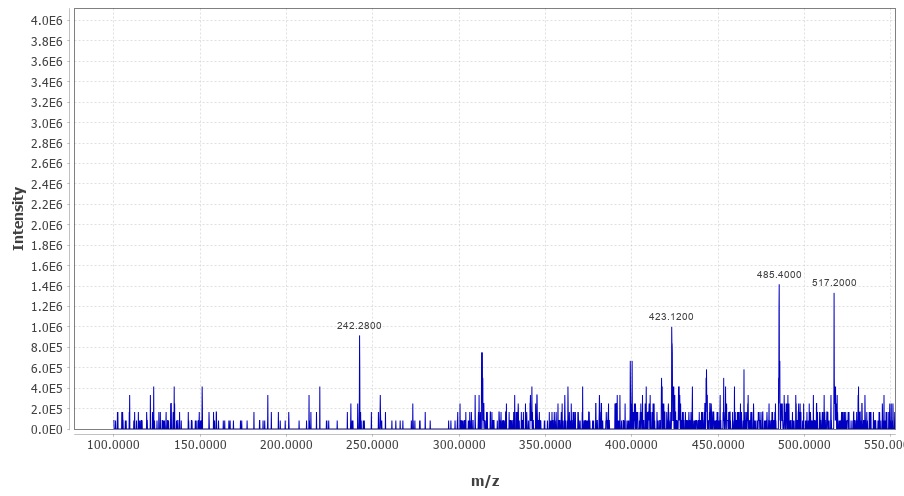

Supplement: S1 File — (DOCX) [file pone.0287071.s001.docx]
