# Supplementary material for: Tamarix articulata extract offers protection against toxicity induced by beauty products in Hs27 human skin fibroblasts
Source: PLoS One. 2023 Nov 16;18(11):e0287071. doi: 10.1371/journal.pone.0287071 (PMC10653522; doi:10.1371/journal.pone.0287071)
Supplement: S1 Raw image — (PDF) [file pone.0287071.s002.pdf]

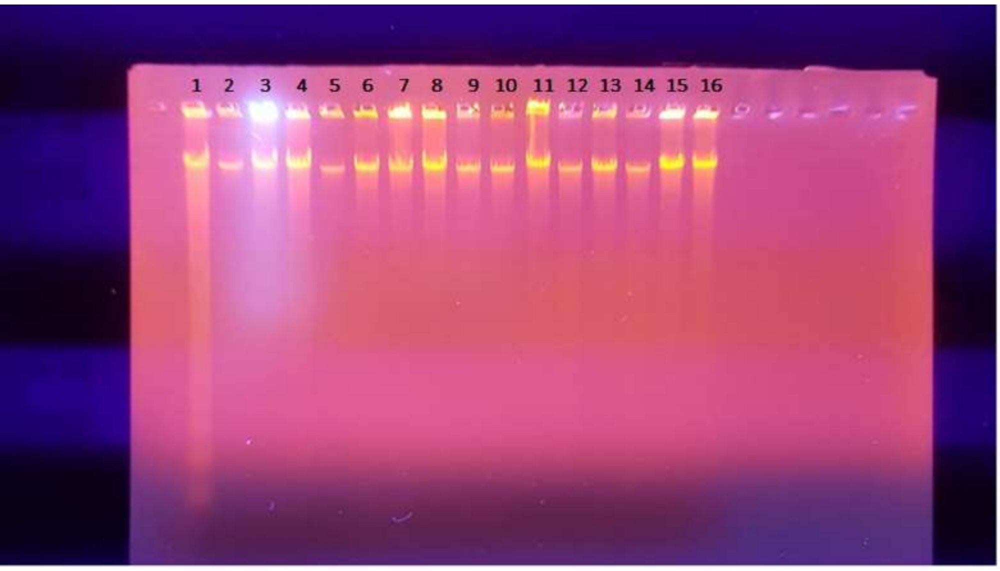

1. Camptothecin (CPT) 10 µM
2. Control (untreated)
3. Hudabeauty liquid matte sample no. 11 (250 µg/mL)
4. Hudabeauty liquid matte sample no. 11 (500 µg/mL)
5. Hudabeauty liquid matte sample no. 10 (250 µg/mL)
6. Hudabeauty liquid matte sample no. 10 (500 µg/mL)
7. Revlon matte sample no. 13 (250 µg/mL)
8. Revlon matte sample no. 13 (500 µg/mL)
9. Revlon matte sample no. 12 (250 µg/mL)
10. Revlon matte sample no. 12 (500 µg/mL)
11. Maybelline New York sample no. 14 (250 µg/mL)
12. Maybelline New York sample no. 14 (500 µg/mL)
13. Beesline apitherapy sample no 16 (250 µg/mL)
14. Beesline apitherapy sample no 16 (500 µg/mL)
15. Maybelline New York sample no. 15 (250 µg/mL)
16. Maybelline New York sample no. 15 (500 µg/mL)
